# Small-Cell Lung Cancer: Is the Black Box Finally Opening Up?

**DOI:** 10.3390/cancers13020236

**Published:** 2021-01-11

**Authors:** Birgitta I. Hiddinga, Klaas Kok

**Affiliations:** 1 Department of Pulmonary Medicine and Tuberculosis, University Medical Center Groningen, 9713 GZ Groningen, The Netherlands; 2 Department of Genetics, University Medical Center Groningen, University of Groningen, 9700 RB Groningen, The Netherlands; k.kok@umcg.nl

Small-cell lung cancer (SCLC) is an aggressive cancer that originates from the neuroendocrine crest. It comprises about 15% of lung cancers and has a dismal prognosis [1]. The 5-year survival has hardly increased in recent decades and is now 6% [1]. In the last decade, few new treatment modalities were implemented, and treatment with chemotherapy and radiotherapy have remained the mainstay of therapy [2]. Thus, in clinical practice we are still in need of targetable biomarkers to treat patients with SCLC in order to substantially improve prognosis in this patient category. In this Special Issue on “Targeted therapy for Small Cell Lung Cancer” we aim to open up the black box that SCLC used to be, trying to reveal targetable biomarkers, as well as biomarkers that can stratify patient groups for more effective treatments, in order to improve the prognosis for this devastating disease.

At the genomic level, SCLC is characterized by a high mutational burden with close to, or even more than, ten somatic mutations per megabase and high chromosomal instability [3,4]. TP53 and RB1 are the most frequently mutated genes in SCLC, resulting in loss of function for these genes [5]. Despite this high mutational burden, very few targetable driver mutations are observed. Mutations in activated tyrosine kinases that can subsequently be targeted by specific inhibitors (TKIs) are a well-known phenomenon for adenocarcinoma of the lung, but are rare in SCLC [6,7]. In the treatment of SCLC, these TKIs have failed. The addition of immunotherapy to chemotherapy in the first line treatment of advanced stage SCLC, instigated by the high mutational burden, improved progression free survival by only two months [8]. Of special interest when treating with immunotherapy, biomarkers such as PDL1 and TMB did not prove as reliable biomarkers to predict the patient population that would benefit from treatment with immunotherapy [9].

Recently, four subsets of SCLC have been described: achaete-scute homolog 1 (ASCL1), neurogenic differentiation factor 1 (NeuroD1), yes-associated protein 1 (YAP1) and POU domain class 2 homebox 3 (POU2F3). These molecular subtypes appear to be associated with distinct expression profiles and have different therapeutical sensitivities [10]. Most cases of SCLC express INSM1, a marker of both ASCL1-high and NEUROD1-high neuroendocrine subtypes and super-enhanced landscapes in SCLC [11]. A small fraction of SCLC tumors are INSM1-low, ASCL1-low and NeuroD1-low. These tumors lack neuroendocrine markers and appear to fall into discrete YAP1-high and POU2F3-high subtypes [12].

## 1. The Search for Novel Targeted Treatments

Both Schultze et al. [13] and Lum et al. [14] give a general overview of the attempts that have been pursued in the past years to develop new targeted therapies for SCLC, although with slightly different angles of approach. After introducing the mutational profile that characterizes SCLC, Schulze et al. [13] describe why current diagnostic tools have not been successful. Subsequently they present a broad perspective on possible future options for molecular-targeted therapy, especially focusing on potential biomarkers for treatment. The authors highlight several surface markers, apoptotic markers, genetic alterations and vascular targets that may act as promising targets and review the status of a number of clinical and preclinical studies. Some of the targeted treatments did not improve the outcome and have already been withdrawn. Several additional novel targets need further evaluation and prospective validation before entering the clinic. In their opinion, the data on aurora kinase inhibitors and PARP inhibitors present the most promising developments. Aurora kinases phosphorylate key components of the cell cycle, whereas PARP1 is an important enzyme in DNA damage repair pathways.

Lum et al. [14] review the genomic structure of SCLC and describe the further subclassification of SCLC into four distinct molecular subtypes: ASCL1, NeuroD1, YAP1 and POU2F3, for which there may be distinct therapeutic targets. They also address an additional complicating factor in the treatment of SCLC, e.g., inter- and intratumor heterogeneity. Intertumor heterogeneity, also a reflection of the above-mentioned subtyping, demands for the efficient stratification of patients with respect to the available treatment strategies. Intratumor heterogeneity may result in not all tumor cells of a patient responding equally to the same treatment. In the absence of oncogenic driver gene mutations that can be targeted, many strategies focus on interfering with crucial pathways and cellular processes. In their extensive overview, Lum et al. group the current therapeutic strategies into five major target categories: development and regulatory pathways, DNA damage and repair (DDR) pathways, epigenetic processes, cell cycle, and the immunotherapy field. Evolving classification of SCLC molecular subtypes is being anticipated as a major development and may help to further stratify patients. In the future, efficient treatment of patients will also depend on the availability of biomarkers that efficiently select patients that respond to a specific treatment. In this respect, real future-changing perspectives will be expected in the field of liquid biopsies. Liquid biopsy analyses could potentially help to stratify patients, for longitudinal monitoring of disease, and for early detection of progression. As reviewed by Lum et al., for SCLC, the focus has been on circulating tumor cell counts and the analysis of circulating cell free DNA. The authors conclude that improved understanding of this devastating disease is underway to underpin the genomics, molecular profiling, resistance mechanisms, and novel therapies in SCLC. They see the further subclassification of SCLC as a major and important step in the development of personalized targeted therapies, but at the same time recognize the high mutation rate and the intratumor heterogeneity as threat that needs to be dealt with.

## 2. Targeting Replication Stress

A characteristic feature of SCLC is the high level of replication stress (RS), most likely correlated with the high mutation rate. A high level of RS can cause mitotic catastrophe and ultimately cell death [15]. To counteract this, the tumor cells need a very robust DNA damage response (DDR) system or a very active replication stress response (RSR) pathway. These might be considered as SCLC’s weak spots. After providing a short review on the principles of RS, Bian et al. [16] summarize the source of RS in SCLC and review the strategies to take advantage of this by either increasing the RS or blocking the DDR system in the tumor cells. Of several inhibitors that target the replication stress pathway, PARP1 and WEE1 appear to be the most promising, based both on preclinical and clinical studies. DNA repair targets encompass ATM, RAD51, but also several genes involved in cell cycle control such as PLK1 and aurora kinases. Although several of the inhibitors developed against these genes have shown some effect as monotherapies, they may be even more effective in combination with a second therapy. Drug-resistance is often due to the complexity of the DNA damage response network, so combinations of replication stress inducers with other therapeutics are explored. As an example, Bian et al. review how targeting PARP appears to increase the sensitivity towards immunotherapy by increasing PD-L1 expression.

## 3. Targeting the Mesenchymal–Epithelial Transition (MET) Pathway

Yet another angle to develop new therapies is discussed by Harby-Werbin et al. [17]. They review the role of the hepatocyte growth factor (HGF)/mesenchymal–epithelial transition (MET) pathway, the activation of which is, amongst others, associated with chemoresistance in SCLC. In metastasized SCLC, overexpression of nuclear topoisomerase-1 correlated with an increased overexpression of MET. As a proof-of-concept, patients with SCLC were treated with MET inhibitors. Although the outcome of these studies suggested a role for MET inhibition in SCLC, in clinical trials the successes have been disappointing. As MET is acting as an epitope, it could be recognized by cytotoxic CD8+ T cells, eliciting an activation toward tumor cells expressing MET. This is the rationale for combination therapy of a MET-inhibitor with immunotherapy. The authors indicate the importance of assessing the tumor tissue for at least MET-expression and/or MET-mutations, HGF expression and immune infiltrate evaluation as potential biomarkers to select patients that will profit from a combined treatment of MET inhibitors and immunotherapy.

## 4. Focal Adhesion Kinase Inhibition

In an original article on the expression and activation of focal adhesion kinase (FAK) Aboubakar Nana et al. [18] evaluated the potential role of FAK as a prognostic marker. FAK is a tyrosine kinase found in focal adhesions, intracellular complexes that are formed following engagement of the extracellular matrix by integrins. FAK-dependent activation of several downstream pathways has been implicated in multiple cellular processes, including cell migration, growth factor signaling, cell cycle progression and cell survival. Consistent with its role in cell migration and angiogenesis, increased abundance or activation of FAK is found in a variety of cancers. As the prevalence of FAK expression in lung cancers was unknown, the authors analyzed 95 non-small cell lung carcinoma (NSCLC), 105 SCLC and 37 normal lung tissue specimens for FAK and phospho-FAK protein levels. FAK abundance was significantly higher in the more malignant SCLC than in NSCLC, which in turn had significantly higher expression levels than normal lung tissue. The phosphor-FAK fraction appeared to be significantly higher in SCLC as compared to NSCLC as well, both in the cytoplasmic and the nuclear compartment. However, neither the expression level of FAK nor that of phospho-FAK correlated with recurrence-free and overall survival in NSCLC and SCLC patients. The authors conclude that FAK-expression cannot act as a prognostic biomarker in SCLC but may be interesting in terms of targeted therapy. However, FAK could still be a biomarker to select patients that may respond to FAK inhibitors.

This latter option is further explored in a review by Aboubakar Nana et al. [19], that starts out with an in-depth description of the role of FAK in multiple cellular processes and how its overexpression and activation is a recurrent factor in solid tumors. As silencing of FAK augments apoptosis in cancer cells, synergistic effects of combinations with other therapies like chemotherapy, immunotherapy or radiotherapy may improve patient outcomes. Of special interest, is the role of FAK in immunotherapy as FAK activity was elevated and correlated with high levels of fibrosis and poor CD8+ cytotoxic T cell infiltration. FAK inhibition substantially limited tumor progression, resulting in a delay in tumor progression, associated with markedly reduced tumor fibrosis and a decreased number of tumor-infiltrating immunosuppressive cells. This raises the question whether FAK inhibition added to immunotherapy is able to result in a better prognosis in SCLC. The reviews discussed above have all shown some promising preclinical results with respect to targeted therapy. However, in subsequent clinical studies these therapies often show disappointing results.

## 5. Drug Delivery Systems

A major hurdle is delivery of the drug to the tumor cells without causing detrimental damage to healthy tissues. Tumor-targeting drug conjugates are an emerging novel therapeutic approach in SCLC, and in their review Deneka et al. discuss the possibilities and ongoing studies regarding delivery systems to bring therapy into the vicinity of the tumor [20]. Antibody–drug conjugates (ADC), radioimmunoconjugates (RICs), small molecule–drug conjugates (SMDCs) and polymer–drug conjugates (PDCs) are the major delivery systems. Several benefits are clear: better prevention of off-target toxicity and trying to allow an effective dosage of the drug to the tumor with a tolerable toxicity. A challenge is to elude systemic toxicity to optimize the response on the tumor. All options need biomarkers to target for effective use of drug conjugates. Several questions are of importance to the clinician. Can we deliver these targeted treatments together with radiotherapy, or in combination with other drugs, for example immunotherapy? In the near future, radionuclides linked to tumor-targeting antibodies may play a prominent role in tumor imaging and optimizing the diagnostic path that patient will follow. Limitations are in the nature of their radioactive payloads.

## 6. Concluding Remarks

In reading the contributions to this special issue, one might conclude the glass is half empty, and that the main conclusion is that SCLC is a resilient tumor for which treatment options are still distant. Although a number of preclinical trials have shown exiting results, subsequent clinical trials have often yielded disappointing outcomes. On the other hand, one could also conclude that the glass is half full, as an enormous amount of effort has been dedicated to these studies, and that recent studies do show progress in finding better treatments for this disease. Some new targets have been identified, with the aurora kinases as good example, that could potentially lead to better treatments. It could well be that it is not a shortage of therapeutic targets that is the main problem, but instead the knowledge on how and when to use them. Three main directions that emerge from the contributions could potentially improve outcomes in patients with SCLC. Firstly, we need more tools to accurately stratify patients for specific treatments. The intratumor heterogeneity may be the main problem in the treatment of SCLC, as it results in no single treatment being effective for the majority of patients. The outcome of clinical studies may improve considerably if tools were available to carefully preselect patients that could potentially benefit from a specific treatment. The further classification of SCLC into four subgroups is a first step in this process [12]. MYC amplification has already been suggested to predict sensitivity towards aurora kinase inhibitors [21]. Secondly, the solution may be lying in combination therapy rather than monotherapy. Targeting dual pathways can be more efficacious to stop proliferation of tumor cells. The included reviews give ample suggestions where a novel drug may actually have a synergistic effect on the response to one of the classic treatments. Thirdly, novel drug delivery systems should help to specifically target the tumor cells while preventing deleterious effects due to the interaction with healthy cells. Taken together in this Special Issue, several routes have been discussed that may lead to new promising treatments for SCLC.

## References

[1] Sabari J.K., Lok B.H., Laird J.H., Laird J.H., Poirier J.T., Rudin C.M. (2017). Unravelling the biology of SCLC: Implications for therapy. Nat. Rev. Clin. Oncol..

[2] Früh M., De Ruysscher D., Popat S., Crinò L., Peters S., Felip E., ESMO Guidelines Working Group (2013). Small-cell lung cancer (SCLC): ESMO Clinical Practice Guidelines for diagnosis, treatment and follow-up. Ann. Oncol..

[3] Murphy J.M., Rodriguez Y.A.R., Jeong K., Ahn E.E., Lim S.S. (2020). Targeting focal adhesion kinase in cancer cells and the tumor microenvironment. Exp. Mol. Med..

[4] Lee S.H., Lee B., Shim J.H., Lee K.W., Yun J.W., Kim S.-Y., Kim T.-Y., Kim Y.H., Ko Y.H., Chung H.C. (2019). Landscape of actionable genetic alterations profiled from 1071 tumor samples in Korean cancer patients. Cancer Res. Treat..

[5] George J., Lim J.S., Jang S., Cun Y., Ozretić L., Kong G., Leenders F., Lu X., Fernández-Cuesta F., Bosco G. (2015). Comprehensive genomic profiles of small cell lung cancer. Nature.

[6] Maemondo M., Inoue A., Kobayashi K., Suguwara S., Oizumi S., Isobe H., Gemma A., Harada M., Yoshizawa H., Kinoshita I. (2010). Gefitinib or chemotherapy for Non-small-cell lung cancer with mutated EGFR. N. Engl. J. Med..

[7] Solomon B.J., Mok T., Kim D.-W., Wu Y.-L., Nakagawa K., Mekhail T., Felip E., Cappuzzo F., Paolini J., Usari T. (2014). First-Line Crizotinib versus Chemotherapy in ALK-Positive Lung Cancer. N. Engl. J. Med..

[8] Horn L., Mansfield A.S., Szczęsna A., Havel L., Krzakowski M., Hochmair M.J., Huemer F., Losonczy G., Johnson M.L., Nishio M. (2018). First-line atezolizumab plus chemotherapy in extensive-stage small-cell lung cancer. N. Engl. J. Med..

[9] Ishii H., Azuma K., Kawahara A., Yamada K., Imamura Y., Tokito T., Kinoshita T., Kage M., Hoshino T. (2015). Significance of programmed cell death-ligand 1 expression and its association with survival in patients with small cell lung cancer. J. Thorac. Oncol..

[10] Rudin C.M., Poirier J.T., Byers L.A., Dive C., Dowlati A., George J., Heymach J.V., Johnson J.E., Lehman J.M., MacPherson D. (2019). Molecular subtypes of small cell lung cancer: A synthesis of human and mouse model data. Nat. Rev. Cancer.

[11] Borromeo M.D., Savage T.K., Kollipara R.K., He M., Augustyn A., Osborne J.K., Girard L., Minna J.D., Gazdar A.F., Cobb M.H. (2016). ASCL1 and NEUROD1 Reveal Heterogeneity in Pulmonary Neuroendocrine Tumors and Regulate Distinct Genetic Programs. Cell Rep..

[12] Baine M.K., Hsieh M.-S., Lai V., Egger J.V., Jungbluth A.A., Daneshbod Y., Beras A., Spencer R., Lopardo J., Bodd F. (2020). SCLC Subtypes Defined by ASCL1, NEUROD1, POU2F3, and YAP1: A Comprehensive Immunohistochemical and Histopathologic Characterization. J. Thorac. Oncol..

[13] Schulze A.B., Evers G., Kerkhoff A., Mohr M., Schliemann C., Berdel W.E., Schmidt L.H. (2019). Future options of molecular-targeted therapy in small cell lung cancer. Cancers.

[14] Lum C., Alamgeer M. (2019). Technological and therapeutic advances in small cell lung cancer. Cancers.

[15] Byers L.A., Rudin C.M. (2015). Small cell lung cancer: Where do we go from here?. Cancer.

[16] Bian X., Lin W. (2019). Targeting DNA replication stress and DNA double-strand break repair for optimizing SCLC treatment. Cancers.

[17] Hardy-Werbin M., Del Rey-Vergara R., Galindo-Campos M.A., Moliner L., Arriola E. (2019). MET inhibitors in Small Cell Lung Cancer: From the bench to the bedside. Cancers.

[18] Aboubakar Nana F., Hoton D., Ambroise J., Lecocq M., Vanderputten M., Sibille Y., Vanaudenaerde B., Pilette C., Bouzin C., Ocak S. (2019). Increased expression and activation of FAK in small-cell lung cancer compared to non-small-cell lung cancer. Cancers.

[19] Aboubakar Nana F., Vanderputten M., Ocak S. (2019). Role of focal adhesion kinase in small-cell lung cancer and its potential role as a therapeutic target. Cancers.

[20] Deneka A.Y., Boumber Y., Beck T., Golemis E.A. (2019). Tumour-targeted drug conjugates as an emerging novel therapeutic approach in small cell lung cancer. Cancers.

[21] Mollaoglu G., Guthrie M., Böhm S., Brägelmann J., Can I., Ballieu P.M., Marx A., George J., Heinen C., Chalishazar M.D. (2017). MYC drives progression of small cell lung cancer to a variant neuroendocrine subtype with vulnerability to aurora kinase inhibition. Cancer Cell.

